# Parasites of the Reintroduced Iberian Lynx (*Lynx pardinus*) and Sympatric Mesocarnivores in Extremadura, Spain

**DOI:** 10.3390/pathogens10030274

**Published:** 2021-03-01

**Authors:** Ana M. Figueiredo, Luís Madeira de Carvalho, María J. P. González, Rita T. Torres, Samuel Pla, Juan C. Núñez-Arjona, Carmen Rueda, Núria Vallverdú-Coll, Fernando Silvestre, Jorge Peña, David Carmena, Miguel A. Habela, Rafael Calero-Bernal, Carlos Fonseca, Fernando Nájera

**Affiliations:** 1 Department of Biology and CESAM, University of Aveiro, Campus Universitário de Santiago, 3810-193 Aveiro, Portugal; rita.torres@ua.pt (R.T.T.); cfonseca@ua.pt (C.F.); 2 CIISA—Centro de Investigação Interdisciplinar em Sanidade Animal, Faculdade de Medicina Veterinária, Universidade de Lisboa, Avenida da Universidade Técnica, 1300-477 Lisboa, Portugal; madeiradecarvalho@fmv.ulisboa.pt; 3 DGMA, Junta de Extremadura Consejería de Medio Ambiente y Rural, Políticas Agrarias y Territorio, Avda. Luis Ramallo S/N, 06800 Mérida, Badajoz, Spain; mariajesus.palacios@juntaex.es (M.J.P.G.); jorgepmartinez@gmail.com (J.P.); 4 Fundación CBD-Hábitat, c/Gustavo Fernández Balbuena 2, Entreplanta, Oficina A, 28002 Madrid, Spain; samuel.pla@cbd-habitat.com (S.P.); carmen.rueda91@gmail.com (C.R.); f.silvestre@cbd-habitat.com (F.S.); 5 Tragsatec, Gerencia de Calidad, Evaluación Ambiental y Biodiversidad, C/Julián Camarillo 6B, Planta 4, 28037 Madrid, Spain; jcnarjona@yahoo.com; 6 FOTEX-Dirección General de Medio Ambiente, Junta de Extremadura, Avda. Luis Ramallo s/n, 06800 Mérida, Badajoz, Spain; nuriavcoll@hotmail.com; 7 Parasitology Reference and Research Laboratory, Spanish National Centre for Microbiology, Majadahonda, 28220 Madrid, Spain; dacarmena@isciii.es; 8 Parasitology Area, Animal Health Department, University of Extremadura, Avda. de la Universidad s/n, 10003 Cáceres, Spain; mahabela@unex.es; 9 SALUVET, Animal Health Department, Faculty of Veterinary Sciences, Complutense University of Madrid, Ciudad Universitaria s/n, 28040 Madrid, Spain; r.calero@ucm.es; 10 Asistencia Técnica de la Dirección General del Medio Natural y Desarrollo Sostenible de la Junta de Comunidades de Castilla-La Mancha, Plaza del Cardenal Siliceo s/n, 45071 Toledo, Spain; 11 Department of Animal Physiology, Faculty of Veterinary Medicine, Complutense University of Madrid, Avda, Ciudad Universitaria s/n, 28040 Madrid, Spain

**Keywords:** Iberian lynx, mesocarnivores, parasites, Ancylostomatidae, *Toxocara cati*, *Trichuris* sp.

## Abstract

The Iberian lynx (*Lynx pardinus*) is one of the most endangered felid species in the world. Conservation efforts have increased its population size and distribution and reinforced their genetic diversity through captive breeding and reintroduction programmes. Among several threats that the Iberian lynx faces, infectious and parasitic diseases have underlined effects on the health of their newly reintroduced populations, being essential to identify the primary sources of these agents and assess populations health status. To achieve this, 79 fresh faecal samples from Iberian lynx and sympatric mesocarnivores were collected in the reintroduction area of Extremadura, Spain. Samples were submitted to copromicroscopic analyses to assess parasite diversity, prevalence, and mean intensity of parasite burden. Overall, 19 (24.1%, ±15.1–35.0) samples were positive for at least one enteric parasite species. Parasite diversity and prevalence were higher in the Iberian lynx (43.8%) compared with the others mesocarnivores under study (e.g., the red fox *Vulpes vulpes* and the Egyptian mongoose *Herpestes ichneumon*). Ancylostomatidae and *Toxocara cati* were the most prevalent (15.6%) parasites. Obtained results revealed that Iberian lynx role as predator control might have reduced parasite cross-transmission between this felid and mesocarnivores due to their decreasing abundances. Surveillance programs must include regular monitoring of this endangered felid, comprising mesocarnivores, but also domestic/feral and wild cat communities.

## 1. Introduction

The Iberian lynx (*Lynx pardinus*), an endemic species of the Iberian Peninsula, is considered one of the most threatened Felidae species in the world, listed as “endangered” by the IUCN Red List of Endangered Species [1]. Since the beginning of the XXI century, in situ and ex situ conservation programs have been carried out in Spain and Portugal, increasing their population size, expanding their distribution area, and reinforcing their genetic diversity through captive breeding and reintroduction programmes [2]. Despite the conservation efforts carried out to prevent the extinction of this species, the continuous effects of habitat destruction and fragmentation, road kills, illegal trapping/hunting, low densities of its main prey—the European wild rabbit (*Oryctolagus cuniculus*)—and infectious diseases are still threatening the survival and thrive of this species [3,4,5,6]. Among the abovementioned threats, infectious diseases have been in the spotlight in recent decades, either due to the decrease in the European wild rabbit populations affected by the myxomatosis and the rabbit haemorrhagic disease (RHD) or by their direct effects on the health of Iberian lynx populations; e.g., feline leukaemia virus (FeLV), Aujeszky’s disease, sarcoptic mange, tuberculosis, and feline parvovirus infections [5,7,8,9,10,11] are among the main reported diseases. Furthermore, the infectious and parasitic diseases mentioned above are of major importance given parasites key role in ecosystem balance and health [11,12] and potential impact on the community structure [13]. Previous studies performed on the Iberian lynx population at Doñana National Park, Sierra Morena and Montes de Toledo [14,15,16,17,18], in Central and Southern Spain, reported different degrees of infection and prevalence of several helminths (e.g., *Toxocara cati*, *Toxocara canis*, *Toxascaris leonina*, *Ancylostoma* spp., *Eucoleus aerophilus*, *Taenia* spp., *Hymenolepis* spp., *Mesocestoides* sp.). Considering its potential impact on host physical condition and population stability, the study of parasite burdens is utterly essential for management and conservation purposes of Iberian lynx populations [14,16,18].

As an apex predator, the restoration of this feline in areas of historical presence has had a negative impact on sympatric mesocarnivores densities (e.g., the red fox *Vulpes vulpes*, the Egyptian mongoose *Herpestes ichneumon*, and the common genet *Genetta genetta*), given the Iberian lynx’s aggressive behaviour towards other carnivores and well-established territories [19]. This behavioural trait of the Iberian lynx has been proved in a quasi-experimental assessment in Extremadura (Spain) between 2014, coinciding with the beginning of the reintroduction program, and 2018, where a total of 38 lynxes had already been released in that area. This reintroduced population played an important role as predator control, having drastically reduced mesocarnivores abundances since the beginning of the reintroduction program [19]. However, their encounters and interactions can increase the risk of lynx exposure to pathogenic agents [20]; e.g., Mateo et al. [21], Santín et al. [22], and Calero-Bernal et al. [23] recently reported the infection of the protist species *Giardia duodenalis*, *Cryptosporidium* spp., *Enterocytozoon bieneusi*, and *Blastocystis* spp. in mesocarnivore communities in overlap areas of the Iberian lynx presence, being crucial to assess and monitor the health status of both the Iberian lynx and sympatric species [7].

Hereupon, this study aims to (i) identify and determine the prevalence and intensity of the enteric parasitic fauna present in the recently reintroduced Iberian lynx population and the sympatric mesocarnivores community of Extremadura region; (ii) understand the potential of parasite cross-transmission between the mesocarnivore reservoirs and the Iberian lynx, as they belong to the same carnivore guild. To our knowledge, this is the first study performed, using conventional copromicroscopic methods, to assess the parasitic fauna in this newly reintroduced Iberian lynx population.

## 2. Results

From a total of 79 samples, 19 (24.1%, ±15.1–35.0) were infected with four different helminths and one protozoa (Table 1). Helminth eggs and protozoa oocysts were identified according to Thienpont et al. [24] and Zajac and Conboy [25]: Ancylostomatidae eggs were determined based on size, ovoid shape, thin and smooth walls, and large blastomeres; *Toxocara* cati and *Toxascaris leonina* eggs were differentiated based on the size (*T. leonina* eggs are larger, measuring ca. 75 × 85 μm), colour, and structure: *T. leonina* presents a thick, smooth and colourless shell, with a yellowish-brown content that only occupies part of the shell, which differs from *T. cati* egg, which displays a thick, rough, and pitted shell with a dark brown content that occupies all the compartment; *Trichuris* sp. was identified based on its size (70–90 μm × 32–41 μm), lemon shape, transparent convex polar plugs, and thick shell with a smooth surface [24]; *Cystoisospora* spp. oocysts were determined based on its elliptical shape, smooth, clear cyst walls, and the presence of a single, round cell (sporoblast) [25]. In total, 43.8% (14/32) Iberian lynx samples were positive for at least one parasite, while in the red fox and the Egyptian mongoose, 14.3% (3/21) and 5.6% (1/18) of the analysed samples showed parasitic forms, respectively. The only stone marten (*Martes foina*) sample analysed was also positive with one parasitic form. No enteric parasites were detected on the samples collected from the genet, the Eurasian otter (*Lutra lutra*) or the European badger (*Meles meles*). All the parasites found were identified with the Willis flotation technique, which is suitable to identify nematode eggs/coccidia oocysts like the ones we found. The same parasites were also found in the same parasitized samples after performing the sedimentation technique, even though this technique is normally used to identify trematode eggs. Most of the infections were monospecific, whereas mixed infections were only present in Iberian lynx samples, with a total of four (12.5%) samples presenting more than one parasitic form; two samples (6.3%) presented *T. cati* and *T. leonina*, and another two (6.3%) samples presented *Trichuris* sp., *T. cati* and *Cystoisospora* sp. oocysts. No L1 of lungworms nematodes were detected with the Baermann technique.

We did not find any significant differences for Ancylostomatidae, a parasite found in the Iberian lynx and another three mesocarnivores (even though the stone marten was not considered for statistical purposes given the low sample size) (χ2 = 1.3, df = 2, *p* = 0.53).

Table 2 shows the mean intensity (EPG) and the respective parasite ranges obtained with the McMaster quantitative technique, which has a minimum threshold of 50 eggs/oocysts per gram of faeces [24,25]. *Toxocara cati* was the one with the highest EPG (4566.7), followed by *Cystoisospora* sp. (1250), both found in Iberian lynx samples. These EPG results show that the parasites present in Table 2 were not spurious infections found in the analysed samples.

## 3. Discussion

All the parasites found in this study have been previously reported in Iberian lynx [14,15,16,17,18] except for *Trichuris* sp. which, to our knowledge, was the first description in this wild felid. Notwithstanding, *Trichuris felis*, *Trichuris campanula*, and *Trichuris serrata* were previously reported in cats [25,26] and *T. vulpis* in several wild carnivores [27]. Nonetheless, considering that our study is only based on morphological identification, we cannot exclude the possibility of *Trichuris* sp. in Iberian lynx as a pseudoparasitism event, acquired from the ingestion of an infected host, despite the high EPG found in one of the samples (200 EPG).

Previous studies recorded a higher prevalence of *Ancylostoma* sp. (22.2% Rodríguez and Carbonell [14], 24.2% Acosta et al. [18], and 57.8% Vicente et al. [16]), comparatively, with the one we found in the Iberian lynx. *Ancylostoma tubaeforme* was previously found with a lower prevalence (12.5%) [15], despite their lower sample size (n = 8). The abovementioned authors also reported a higher prevalence of *T. cati*, *T. leonina*, and *Cystoisospora felis* (e.g., Acosta et al. [18] reported a prevalence of 19.7% for *Toxocara* sp. and 16.7% for *T. leonina*; Rodríguez and Carbonell [14], 33.3% for *T. leonina*, 22.2% for *C. felis*, but a lower prevalence of *T. cati* (11.1%)).

In the red fox, *Cystoisospora canis* was reported by Rodríguez and Carbonell [14] with a higher prevalence (45.0%) than the one we found, but lower for *Ancylostoma* sp. (5.0%). Other authors reported higher prevalence for *Cystoisospora* sp. (e.g., 23.6%, [28]) and for the nematode of the Ancylostomatidae family, *Uncinaria stenocephala*, (e.g., 20.4%, [29]), than the one found in this study for the same nematode family. The nematode *Ancylostoma* sp. was also previously reported in stone marten with a prevalence of 5% [14], and, to our knowledge, this was the first description of Ancylostomatidae parasite in the Egyptian mongoose. *Taenia* spp., an obligate parasite species of carnivores, was not detected in any analysed sample and was previously reported by the abovementioned authors [14,15,17,18]. For example, *T. pisiformis*, reported by Torres et al. [15] with a prevalence of 12.5%, uses rabbits as intermediate host (Iberian lynx main prey) and Canidae/Felidae species as definitive hosts. However, Torres et al. [15], Millán and Casanova [17], and Acosta et al. [18] used lynx carcasses instead of fresh faecal samples as we used in this study, increasing the possibility of finding a greater parasite diversity with higher prevalence, as reported. Furthermore, even though *Taenia* spp. eggs are detected by the Willis flotation technique, they usually passed from the definitive host to the intermediate host in tapeworm segments, and therefore, faecal flotation tends to be a low indicator of the infection status. Likewise, European wild rabbit populations suffered a significant reduction in the last decade due to RHD and Myxomatosis [3], and the absence of Taeniidae infection in the Iberian lynx may be explained by the reductions in this lagomorph populations.

No L1 of lungworms nematodes were detected in the Iberian lynx and mesocarnivores, even though previous studies have reported *Aelurostrongylus abstrusus, Oslerus rostratus, Angiostrongylus chabaudi*, and nematodes of the genus *Troglostrongylus* in wild/domestic cats and other wild Felidae species [30]. *A. abstrusus* was previously recorded in the Eurasian lynx (*Lynx lynx*) with a prevalence of 17% [31].

Average intensity for EPG found on Iberian lynx was high, especially for *T. cati* and *Cystoisospora* sp., but no infection rate was possible to calculate for Ancylostomatidae, which was previously reported by Vicente et al. [16] for *Ancylostoma* sp. with a maximum of 21,195 EPG.

Overall, the prevalence and parasite richness found was somewhat lower than expected, especially in the mesocarnivore community, though the high infection rates found. The parasite Ancylostomatidae was the only one found in both the Iberian lynx and in three mesocarnivore species. Similar to this parasite, *Trichuris* sp., *T. cati*, *T. leonina*, and *Cystoisospora* sp. have direct life cycles (through ingestion of infective eggs, the first three, or oocysts, the fourth), even though the last three agents can also adopt a facultative indirect life cycle (through the ingestion of infected paratenic hosts) [32]. This means that its transmission to other wild hosts do not rely exclusively on an intermediate host and can easily occur through accidental ingestion of eggs or percutaneous contact in contaminated water, soil, faeces, or food [33] or even direct contact with other infected hosts, such as domestic/feral and wild cats. In this regard, Vicente et al. [16] and Millán and Casanova [17] previously informed that cats might play an important role in the life cycle of some parasites, namely the ones with a direct life cycle (*A. tubaeforme* and *T. cati*). This may be an indicator of overlapping between sylvatic and domestic (or peri-domestic) transmission cycles of these parasites, as previously suggested by Barrera et al. [34] for the protist species *Cryptosporidium hominis* found in red foxes in Galicia. Millán and Casanova [17] found an overall higher parasite prevalence in feral cat samples (84%) than in Iberian lynx samples and several parasites that both felines share, which means that domestic/feral and wild cats may be an important reservoir of parasites that can affect the Iberian lynx. In fact, Nájera et al. [35] reported, for the same population in study, a case of interspecific killing and partial consumption of a feral cat by an Iberian lynx individual, which evidence the disease risk and possible outbreak that this domestic/feral species can represent for the Iberian lynx, especially in small/newly reintroduced populations. With the exception of *T. cati*, which is more cat-specific, according to Ribas et al. [36] and Olmedo et al. [37], most of the parasites found in this study have low host specificity and have mutual wild carnivores’ families, which can be explained by their similar generalist diet composition. Rodríguez and Carbonell [14] also found low prevalence and diversity of parasites among the mesocarnivores analysed, especially in the stone marten, the genet, and the Egyptian mongoose. The low sample size can be another reason behind this scenario for the abovementioned species and the Iberian lynx case, since it is a recently reintroduced population with still a relatively low number of individuals. Mesocarnivores samples were harder to find due to lynx role as predator control, which has drastically reduced mesocarnivores abundances since the beginning of the reintroductions in Extremadura [19]. Even though species with larger geographical ranges, such as the Iberian lynx, may present higher parasite richness—given that they use a wider range of habitats and, therefore, contact with a higher number of intermediate, paratenic, and definitive hosts, at the time of the study, lynx encounters and interactions with other mesocarnivores were less frequent [19], reducing the risk of pathogen cross-transmission through direct contact. Additionally, our low parasite prevalence and richness can be associated with seasonal variation effects in the Extremadura region, such as moisture and, especially, temperature, which has shown a trend towards higher maximum extreme values [38], affecting the abundance and activity of intermediate host and parasite development [39], but also the conservation of the samples collected directly from the ground. Events of parasitic fauna extinction, due to the absence of the required intermediate host [40] or the non-predation of key intermediate species [41], but also host body mass and life-history traits, can be considered other associated factors [42].

The low prevalence and parasite richness found could not be explained by the administration of a topical parasiticide before release, not mentioning that it does not work on cestodes, since selamectin only shows activity over nematodes and arthropods. Although lynxes were treated before being reintroduced and may initially benefit from a lack of parasite burden, this effect only lasted for a short period, as the plasma clearance in the parasiticide used is 30 days and the elimination half-life after topical administration is around eight days in cats ([43]; label information; Stronghold^®^, Zoetis Belgium SA, Louvain-la-Neuve, Belgium). Moreover, not only samples were obtained from reintroduced lynxes, since reproduction and wild-born individuals were recorded during the second and subsequent years of the program.

It is essential to highlight this study’s main limitations, which may be another of the reasons behind the overall low diversity, prevalence, and intensity found: (1) the relatively small sample size and the low number of positive samples made it challenging to conduct statistical analyses and hamper obtaining robust conclusions (e.g., even though we reported Ancylostomatidae in stone marten, we only found one sample of this species, thus excluding it from any statistical analysis; additionally, our low samples size may be the reason behind not finding any significant differences for Ancylostomatidae, reported in four of the seven host species in study); (2) the conventional microscopy-based nature of the study may have underestimated the true prevalence of the investigated parasites (particularly protist species), as microscopy is less sensitive than molecular techniques (PCR and sequencing). Previous studies carried out with meso and apex carnivores reported different protist species and prevalence in several regions of Spain (e.g., Mateo et al. [21] reported *Giardia duodenalis* in stone marten (13%), red fox (8.1%), and wolf (17%), *Cryptosporidium hominis* in badger and *C. felis* and *C. ubiquitum* in red fox; Santín et al. [22] found *Enterocytozoon bieneusi* in the European badger, stone marten, and red fox, in a total of 13.2% of the analysed samples; Calero-Bernal et al. [23] reported a total prevalence of 1.6% of *Blastocystis* sp. found in samples of red fox, common genet, and European polecat); (3) the lack of samples in this study from domestic/feral and wild cats, as previously mentioned, may have decreased the total prevalence found, given their host specificity and transmission cycles, favouring disease transmission between domestic animals and wildlife and increasing disease risk for the Iberian lynx [44] and (4) the lack of samples from herbivore reservoirs, such as the European wild rabbit or ungulate species, may have limited the characterisation of transmission pathways and the identification of other potential sources of infection. Indeed, though not reported, once considered a case of pseudoparasitism, we did find *Eimeria* sp. oocysts in three samples of Iberian lynx, which corroborate the need to account in similar future studies herbivore species, given their important role as intermediate hosts of parasites with complex life cycles.

Even though a small portion of the faecal samples we obtained were through routine check-ups, post-mortem examinations, and road-killed incidents, our sampling was predominantly non-invasive, based on the collection of fresh samples in the environment. This methodology is, however, suitable for wildlife studies, especially when we are working with endangered species/populations, such as the Iberian lynx individuals that inhabit Extremadura. Thus, it is imperative to better understand parasites’ true impacts in the ecosystem and their interactions with different organisms to provide additional information to minimise parasitic transmission and outbreaks especially in endangered populations [45]. Considering that the Iberian lynx is still one of the most threatened felid species in the world, it is crucial to apply safety measures to protect all the individuals, not only from parasitic infections but also from other potential and more harmful pathogens [7] that may jeopardise their populations. Our research highlights the importance of studying the parasitic fauna of an endangered species and the mesocarnivore community who overlap lynx range. This preliminary study can be used as a baseline for the implementation of larger epidemiological studies and diseases surveillance programs. Despite the importance that conventional microscopy-based studies still have, especially in lower-budget studies, future studies should aim to use molecular methods that will improve our understanding of the genetic diversity of parasitic species, as well as their transmission and even zoonotic potential.

## 4. Materials and Methods

### 4.1. Study Area

**Figure 1:**
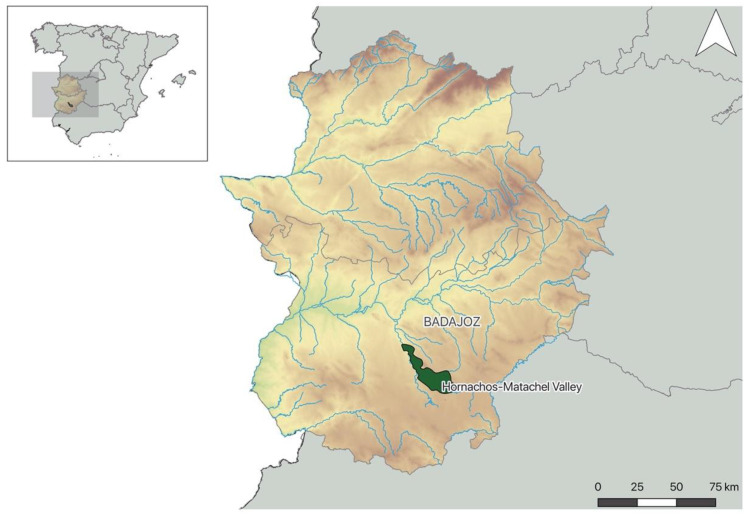
Sample location in Hornachos-Matachel Valley (HMV)—location of the Hornachos-Matachel Valley (HMV, Extremadura, Spain) where the samples from the Iberian lynx and mesocarnivores were collected.

This study was conducted in Hornachos-Matachel Valley (HMV, Extremadura region, Spain) (Figure 1), a study area of the Life + IBERLINCE (LIFE10NAT/ES/570). The habitat is characterized as a Mediterranean scrub, exhibiting a mix between scrublands (e.g., *Erica* spp., *Cistus* spp., and *Rosmarinus* spp.) and dense scrub (*Pistacia lentiscus*, *Quercus coccifera*, *Flueggea tinctorial*), but also open pasture areas. This kind of habitat frequently supports high densities of rabbits, representing 80–99% of Iberian lynx diet [46], since it offers food, refuge, and a suitable structure for the lynx to hunt. Water availability is another essential component in Iberian lynx habitats, as they regularly visit water bodies [47]. Aside from the presence of Mediterranean scrub habitat and numerous water points, the HMV was chosen for reintroduction considering their high rabbit densities across the territory (8.2 rabbits/ha, González, M., personal communication) [48].

### 4.2. Sample Collection and Copromicroscopic Analyses

Lynxes born within the captive facilities of the Iberian lynx Ex-Situ Conservation Program [49], and between two to three weeks before being released, underwent a complete physical examination and infectious disease screening and were vaccinated against feline calicivirus, feline herpesvirus, feline panleukopenia, feline leukaemia viruses, and *Chlamydophila felis* (Fevaxyn Pentofel^®^, Zoetis, Belgium; FeLV PureVAX^®^, Boehringer Ingelheim Vetmedica GmbH, Germany). Additionally, a topical parasiticide, selamectin (Stronghold^®^, Zoetis, Belgium), was administered. All lynxes were radio-collared. Lynxes (reintroduced and wild-born) that were subject to capture after being released (e.g., disease surveillance, radio-collar change, emergency procedures) received a booster of feline leukaemia virus vaccine and depending on ectoparasite (i.e., fleas, ticks, and/or hippoboscid flies) loads, fipronil (Frontline Spot-On Gato^®^, Boehringer Ingelheim Vetmedica GmbH, Germany) was administered topically. For the purpose of this study, between February 2016 and July 2018, 79 fresh faeces were collected from Iberian lynx (n = 32), red fox (n = 21), Egyptian mongoose (n = 18), common genet (n = 3), Eurasian otter (n = 3), European badger (n = 1), and stone marten (n = 1). Samples were collected during routine check-ups/live-capture operations, post-mortem examinations, road-killed incidents, and by prospecting animals’ latrines in several well-distributed transects across the study area (Figure 1). Samples collected from the ground were identified based on their morphology (e.g., shape, size, content of the faeces) and deposition site (animal latrines were confirmed by camera-trapping), by several experienced and field trained personnel, which helped to narrow down the observer error. Collected samples were stored in airtight plastic bags at 4 °C up to a maximum of one month to avoid degradation of parasitic forms until further examination in the laboratory. This conservation method helped to slow down the process of development and hatching of parasite eggs/cysts/oocysts [25].

In each collected sample, parasite eggs/larvae/cysts/oocysts were identified based on size, shape, colour, content, and other key features, according to Thienpont et al. [24] and Zajac and Conboy [25], and their prevalence and mean intensity were evaluated by means of one quantitative technique and three different qualitative techniques: (1) modified McMaster test (quantitative technique), where nematode eggs and coccidian oocysts were counted using a McMaster chamber under a 10× objective; (2) Willis flotation; (3) sedimentation technique use the parasite’s buoyancy and liquid density as segregation factors; these techniques were performed with a saturated sugar solution (3:1) and used to isolate gastrointestinal nematode/cestode eggs and coccidia oocysts, which will float in the solution [24,25], and trematode eggs, the heavier ones, that will sediment. Methylene blue dye was added to the sedimentation slide to help distinguish the eggs from the remaining sediment [50], and preparations were observed under a 10× and 40× objective; (4) modified Baermann technique was used to detect L1 of lungworm nematodes that migrate in a water solution at room temperature [24] within 24 h [24,51]. A slide is then prepared, and first-stage larvae can be observed on a microscope under a 10× and 40× objective and differentiated based on size and the morphology of the oesophagus and tails [25].

### 4.3. Statistical Analysis

Parasite prevalence was calculated based on Bush et al. [52], as the percentage of hosts infected by that parasite species, using a binomial distribution with the function “binom.test” in R software (www.r-project.org), establishing confidence limits within 95% confidence intervals (CI). The intensity of the infection was estimated as the mean number of parasite eggs/oocysts per infected hosts [53], using the McMaster quantitative technique, with a sensitivity of 50 eggs per gram (EPG) of faeces [25]. The χ2-test was calculated using the function “chisq.test” in R software (www.r-project.org) to assess significant differences between parasitic prevalence found in the Iberian lynx and at least one mesocarnivore, for a *p*-value ≤ 0.05.

## 5. Conclusions

The implementation of wildlife disease surveillance programs is crucial for conservation purposes and will allow the identification of disease hotspots to minimise parasitic transmission and outbreaks in the recently reintroduced lynx populations. We suggest that this surveillance programs must include regular monitoring protocols in all Iberian lynx territories of the Iberian Peninsula, comprising the sympatric mesocarnivores communities, but also including domestic/feral cats, and applying, if necessary, preventive or corrective measures.

## Figures and Tables

**Table 1:** Parasite prevalence and confidence intervals (CI, 95%)—number, prevalence, and confidence intervals (CI, 95%) of the parasites found in the Iberian lynx and sympatric mesocarnivores in Extremadura region of Spain.

Species		Iberian Lynx (*Lynx pardinus*)		Red Fox (*Vulpes vulpes*)		Egyptian Mongoose (*Herpestes ichneumon*)		Common Genet (*Genetta genetta*)		Eurasian Otter (*Lutra lutra*)		European Badger (*Meles meles*)		Stone Marten (*Martes foina*)
Total samples		32		21		18		3		3		1		1
**Parasites**	n	% (CI 95%)	n	% (CI 95%)	n	% (CI 95%)	n	% (CI 95%)	n	% (CI 95%)	n	% (CI 95%)	n	% (CI 95%)
**Nematodes**														
Ancylostomatidae	5	15.6 (5.3–32.8)	2	9.5 (1.2–30.4)	1	5.6 (0.1–27.3)	-	-	-	-	-	-	1	NA ^a^
*Toxocara cati*	5	15.6 (5.3–32.8)	-	-	-	-	-	-	-	-	-	-	-	-
*Toxascaris leonina*	4	12.5 (3.5–29.0)	-	-	-	-	-	-	-	-	-	-	-	-
*Trichuris* sp.	3	9.4 (2.0–25.0)	-	-	-	-	-	-	-	-	-	-	-	-
**Protozoa**														
*Cystoisospora* spp.	3	9.4 (2.0–25.0)	1	4.8 (0.1–23.8)	-	-	-	-	-	-	-	-	-	-

^a^ NA—not applied.

**Table 2:** Mean intensities (EPG) and ranges of parasite excretion—mean intensities and ranges of parasitic excretion for the different parasitic infections found in the Iberian lynx and sympatric mesocarnivores, expressed in eggs per gram of faeces (EPG).

Species		Iberian Lynx (*Lynx pardinus*)		Red Fox (*Vulpes vulpes*)		Egyptian Mongoose (*Herpestes ichneumon*)		Common Genet (*Genetta genetta*)		Eurasian Otter (*Lutra lutra*)		European Badger (*Meles meles*)		Stone Marten (*Martes foina*)
Parasites	n	Mean Intensity (Range)	n	Mean Intensity (Range)	n	Mean Intensity (Range)	n	Mean Intensity (Range)	n	Mean Intensity (Range)	n	Mean Intensity (Range)	n	Mean Intensity (Range)
**Nematodes**														
Ancylostomatidae	-	-	1	500 (500)	-	-	-	-	-	-	-	-	-	-
*Toxocara cati*	3	4566.7 (100–11,050)	-	-	-	-	-	-	-	-	-	-	-	-
*Toxascaris leonina*	1	200 (200)	-	-	-	-	-	-	-	-	-	-	-	-
*Trichuris* sp.	1	200 (200)	-	-	-	-	-	-	-	-	-	-	-	-
**Protozoa**														
*Cystoisospora* spp.	2	1250 (100–2400) *	1	50 (50) *	-	-	-	-	-	-	-	-	-	-

* Oocysts.

## Data Availability

Data supporting reported results are available upon request to authors.
